# Endometrial Adenocarcinoma After a Benign Biopsy Without Atypia: A Case Report

**DOI:** 10.7759/cureus.67906

**Published:** 2024-08-27

**Authors:** Samantha DeMartino, Joshua M Keefer, Caleb Huff

**Affiliations:** 1 Obstetrics and Gynecology, West Virginia School of Osteopathic Medicine, Huntington, USA; 2 Surgery, Joan C. Edwards School of Medicine, Marshall University, Huntington, USA; 3 Obstetrics and Gynecology, Valley Health, Huntington, USA

**Keywords:** hysteroscopy, premenopausal, abnormal uterine bleeding, endometrial biopsy, hysterectomy, benign endometrial hyperplasia, endometrial hyperplasia, gynecologic cancer, endometrial adenocarcinoma, hyperplasia without atypia

## Abstract

Endometrial biopsy is a highly effective screening procedure used to determine endometrial cancer and its precursors. This is often used to rule out endometrial cancer, the most common gynecologic cancer in the United States, before a total hysterectomy. This is a case of a benign endometrial biopsy that was ultimately malignant in the post-operative pathology report. A 37-year-old female presents with a six-month history of dysmenorrhea, passage of large clots, and pelvic pain, seeking definitive treatment with a hysterectomy. The pre-operative assessment included ultrasound, hysteroscopic exam, and endometrial biopsy. The ultrasound demonstrated evidence of adenomyosis due to the heterogeneous appearance of the myometrium and an endometrial stripe of 36 mm. Endometrial biopsy using pipelle was performed alongside an in-office hysteroscopic exam, which had a hyperplastic appearance of the endometrium. The biopsy resulted in hyperplasia without atypia and scant polypoid endometrial tissue. The patient underwent a total laparoscopic hysterectomy and bilateral salpingectomy without complications. The post-operative pathology report indicated a grade 2 invasive endometrial adenocarcinoma extending through 75% of the myometrium. Incidental diagnosis of endometrial adenocarcinoma following total hysterectomy is rare and poses significant medical implications. Endometrial hyperplasia without atypia has a low risk of progressing to endometrial carcinoma over time.

## Introduction

Endometrial cancer is the most common gynecological cancer in women, with an expected 66,000 new cases in 2023. This malignancy has a significant impact on females' quality of life, as well as their all-cause mortality. It is also the fourth most common of all malignancies in the female population within the United States [1]. Most cases of endometrial cancer are diagnosed in women older than 50 years [2,3]. This malignancy affects women of all ethnic groups in the United States as well as worldwide, with Black women often being diagnosed at a later stage compared to non-Hispanic White women [1]. There has been an increase in the incidence of this malignancy due to the significant rise in the obesity epidemic in the United States, related to the hyperestrogenic state of obesity [4]. It is estimated that by 2030, there will be a 130% increase in the diagnosis of endometrial carcinoma worldwide.

Endometrioid carcinoma is the most common subclass of endometrial cancer. Endometrial hyperplasia (EH) is a precursor lesion to endometrioid carcinoma and is defined as the abnormal proliferation of the endometrium and its glands lining the uterus. EH is classified into two groups: hyperplasia without atypia and hyperplasia with atypia. Atypia is defined as cells with nuclear enlargement, with the presence or absence of distinct nucleoli. During the normal uterine cycle, estrogen stimulates the proliferation of the endometrium, whereas progesterone stimulates the secretory phase. When estrogen is increased disproportionately to progesterone, there is abnormal proliferation of the uterine lining. Factors that alter the normal physiologic estrogen-to-progesterone ratio can progress to EH with or without atypia, including obesity, consistent anovulatory cycles, and polycystic ovary syndrome (PCOS) [5,6]. Nulliparity, early menarche, late menopause, and older age also increase the risk for EH due to the cumulative effects of excess estrogen exposure [5,6]. The rate of progression is highly variable and depends on a multitude of factors, the most important being the presence of atypia in the hyperplastic endometrium [2,7]. EH without atypia has less than a 5% risk of progression to endometrial carcinoma over 20 years [6,8], whereas studies have shown that up to 50% of women with EH with atypia have occult endometrial carcinoma stage 1 [6].

The most common symptom of EH is abnormal uterine bleeding (AUB). This can present as postmenopausal bleeding and irregular bleeding patterns in premenopausal women. AUB in premenopausal women is most often due to structural causes, including leiomyoma, adenomyosis, and endometrial or cervical polyps; the risk for endometrial carcinoma is unclear [2,3]. Other symptoms of EH include pelvic pain, abdominal cramping, passing large blood clots, and possibly anemia. Abnormal proliferation of the endometrium can also be identified by the presence of atypical glandular cells seen on cervical cytology. Due to the potential risk of occult endometrial carcinoma, symptoms and risk factors suggestive of EH warrant further investigation.

We report a 37-year-old female presenting with AUB, who was diagnosed with stage 2 endometrioid carcinoma after a biopsy that showed EH without atypia.

This article was previously presented as a meeting abstract at the West Virginia School of Osteopathic Medicine Summer Seminar Conference, on June 6, 2024.

## Case presentation

The patient is a 37-year-old, gravid 4, para 4, premenopausal White female who presented to the clinic complaining of constant, AUB for six months. She reported daily bleeding of inconsistent volumes and the passage of large clots. Along with constant bleeding, she complained of daily pelvic pain that radiated toward her lower back. Her medical history includes obesity with a BMI of 37, with no known history of type 2 diabetes, PCOS, or early menarche status.

**Figure 1:**
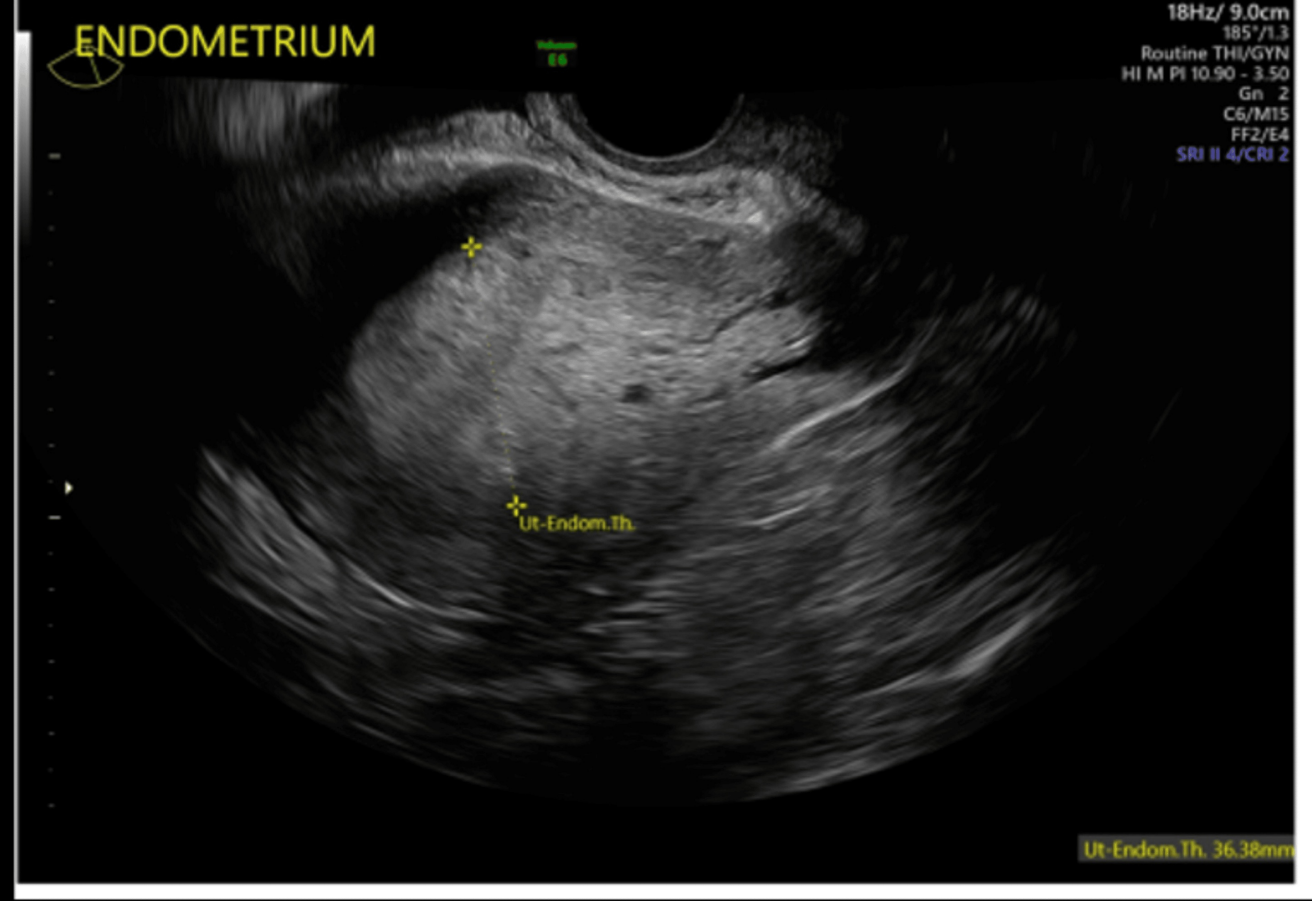
Ultrasound image of the endometrium, demonstrating an endometrial stripe of 36.88 mm.

**Figure 2:**
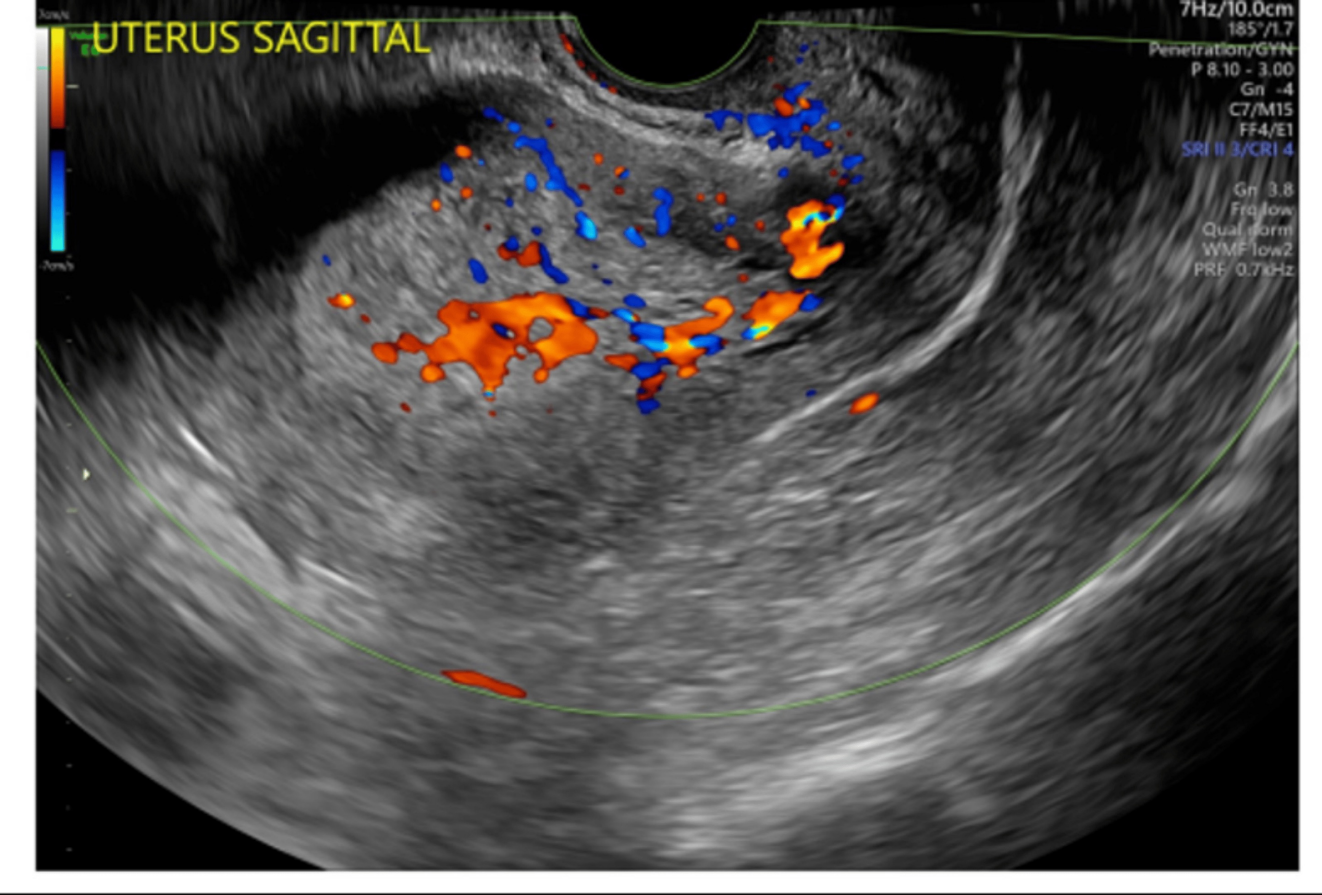
Ultrasound of the uterus, in the sagittal view, showing increased vascularity of the myometrium, suggestive of adenomyosis.

Tranexamic acid was given to help reduce the burden of her abnormal bleeding. She underwent AUB management, including an ultrasound with a Doppler study that demonstrated a thickened endometrial stripe of 36 mm (Figure 1), a heterogeneous myometrium, and increased vascularity (Figure 2), all suggestive of adenomyosis. An endometrial biopsy was performed with a pipelle, a plastic cannula that uses suction to collect the sample, along with a hysteroscopic exam. The endometrium had a hyperplastic and fluffy appearance, and the subsequent biopsy demonstrated scant polypoid endometrial tissue with hyperplasia without atypia.

The following risk factors were considered for endometrial carcinoma in this patient: positive risk factors include obesity and AUB; negative risk factors include young age, premenopausal status, multiparity, and no known history of PCOS, diabetes, or early menarche.

Different treatment options for benign EH were discussed with the patient, such as oral contraceptives, progesterone intrauterine devices, depot medroxyprogesterone acetate (DMPA), Nexplanon, and surgery. Conservative surgical management, such as dilation and curettage and endometrial ablation, was also discussed versus a total hysterectomy. She reported a strong family history of thrombophilia and declined hormonal therapy due to its potential risk. She has four kids and does not desire to preserve fertility. This method of shared decision-making with the patient led us to move forward with a total hysterectomy at the patient’s request.

She underwent a robotic-assisted total laparoscopic hysterectomy with bilateral salpingectomy. The surgeon elected to preserve the ovaries due to her young age and premenopausal status. The patient had an uncomplicated postoperative period and was discharged home. The uterus was sent to pathology for a biopsy, which resulted in a grade 2 invasive endometrioid adenocarcinoma (ETA) with negative margins. The tumor extended through 75% of the myometrium, with a depth of 15 mm. The patient was doing well at her two-week postoperative visit. Her final pathology results were discussed, and she was referred to the Gynecology Oncology Department for further workup and treatment for her grade 2 endometrial adenocarcinoma.

## Discussion

A definitive diagnosis of EH is made with an endometrial sample that shows a proliferation of glandular tissue and an increase in the gland-to-stroma ratio. There are multiple methods of obtaining an endometrial biopsy, the preferred method being hysteroscopy, which allows for direct visualization of the uterus [5,9,10]. The use of hysteroscopy improves biopsy accuracy due to the high likelihood of visualizing a lesion of the uterine lining. Newer literature has shown that hysteroscopy with pipelle biopsy has a higher sensitivity for diagnosing malignancy than dilation and curettage, another technique used for an endometrial biopsy. Detection rates of the biopsy for EH in premenopausal women and postmenopausal women have been reported to be 91% and 99.1%, respectively [5]. Recent studies have shown that benign findings on biopsy have a false negative rate of less than 1% [11]. 

The presence of a gross necrotic or fungating mass seen during hysteroscopy is highly suggestive of endometrial cancer, but a definitive diagnosis requires histologic evaluation via an endometrial biopsy. Our patient’s hysteroscopic exam showed a hyperplastic appearance without evidence of suspicious lesions. We proceeded with an endometrial biopsy using the standard of care, the pipelle method, and collected an adequate sample. Obesity poses difficulties during nearly all gynecologic exams due to vaginal wall collapse covering the cervix, which complicates obtaining samples. The additional complexity from obesity in our patient, with a BMI of 37, could be a potential contributing factor to our false-negative biopsy.

A transvaginal ultrasound is another adjunct tool that can be used in the evaluation of AUB. This can be used in the workup of AUB in postmenopausal women instead of undergoing an endometrial biopsy. It can detect endometrial carcinoma with a sensitivity of 96% if the endometrial thickness is greater than 4 mm. Unfortunately, it cannot be used as an adjunct in premenopausal women due to the continuous change in endometrial thickness during menstrual cycles and the lack of consistent parameters [1,3,5]. Our patient demonstrated an endometrial stripe of 36 mm (Figure 1), but due to her premenopausal status, its significance is undetermined. In addition to her heterogeneous endometrium and thickened endometrial stripe, Doppler imaging showed increased vascularity (Figure 2), which, in conjunction, suggests adenomyosis. Although increased vascularity of the myometrium can also be indicative of a cancerous process, patient presentation and glandular cells on cervical cytology are two factors that physicians use when deciding whether to perform a biopsy in premenopausal women.

Management of EH is determined by the classification of EH, age, and desires of the patient. Treatment options include hormonal therapy, regular surveillance, and surgery. Progestin therapy is recommended for premenopausal women who desire to preserve fertility, regardless of the class of EH [6]. Surveillance is typically not recommended, but it is used for women who cannot take progestin therapy and want to preserve fertility. These patients are monitored closely and undergo endometrial sampling every three months until there are two consecutive negative biopsies [5,6]. Hysterectomy with bilateral salpingo-oophorectomy is curative and recommended in patients with atypical features, as there is a 28% chance of progression to malignancy [6].

This case has important medical implications for the evaluation and management of EH in premenopausal women. Guidelines recommend using age 40 and 45 years old as the cutoff to proceed with a biopsy [2,3]. Current literature does not have a definitive protocol for premenopausal women under the age of 40 with AUB, which may be due to the low incidence. A systematic review found that women with premenopausal AUB have an overall risk for endometrial cancer of 0.33% [2]. This case signifies the need for further research to establish evaluation guidelines in premenopausal women presenting with AUB.

## Conclusions

This case demonstrated a false-negative endometrial biopsy despite the use of hysteroscopy for direct visualization, which occurs in less than 1% of patients. A false-negative biopsy alters the course of management for this population, including a second procedure to complete staging. This case contributes to the very rare diagnosis of endometrial carcinoma after a benign endometrial biopsy in premenopausal women. To our knowledge, no studies have identified stage 2 endometrial carcinoma after receiving a benign biopsy.
